# The New American Joint Committee on Cancer/International Union Against Cancer Staging System for Adenocarcinoma of the Stomach: Increased Complexity without Clear Improvement in Predictive Accuracy

**DOI:** 10.1245/s10434-012-2403-6

**Published:** 2012-05-23

**Authors:** Johan L. Dikken, Cornelis J. H. van de Velde, Mithat Gönen, Marcel Verheij, Murray F. Brennan, Daniel G. Coit

**Affiliations:** 1 Department of Surgery, Memorial Sloan-Kettering Cancer Center, New York, NY USA; 2 Department of Surgical Oncology, Leiden University Medical Center, Leiden, The Netherlands; 3 Department of Epidemiology and Biostatistics, Memorial Sloan-Kettering Cancer Center, New York, NY USA; 4 Department of Radiotherapy, The Netherlands Cancer Institute—Antoni van Leeuwenhoek Hospital, Amsterdam, The Netherlands

## Abstract

**Purpose:**

To evaluate the changes in the 7th edition American Joint Committee on Cancer (AJCC) staging system for stomach cancer compared to the 6th edition; to compare the predictive accuracy of the two staging systems.

**Methods:**

In a combined database containing 2,196 patients who underwent an R0 resection for gastric adenocarcinoma, differences between the two staging systems were evaluated and stage-specific survival estimates compared. Concordance probability and Brier scores were estimated for both systems to examine the predictive accuracy.

**Results:**

Nodal status cutoff values were changed, leading to a more even distribution for the redefined N1, N2, and N3 group. AJCC 6th edition stage II reflected a highly heterogeneous population, which is now adequately subdivided in the AJCC 7th edition into stages IIA, IIB, and IIIA. The predictive accuracy of N classification improved significantly as measured by concordance. Despite increased complexity, the predictive accuracy of AJCC 7th stage grouping was significantly worse than that of the AJCC 6th edition.

**Discussion:**

The increased complexity of the 7th edition staging system is accompanied by improvements in the predictive value of nodal staging as compared to the 6th edition, but it was no better in overall stage-specific predictive accuracy. Future refinements of the tumor, node, metastasis staging system should consider whether increased complexity is balanced by improved prognostic accuracy.

Cancer staging is one of the fundamental activities in oncology.^1^,^2^ For over 50 years, the tumor, node, metastasis staging system (TNM) has been a standard in classifying the anatomic extent of disease.^3^ In order to maintain the staging system relevant, the International Union Against Cancer and the American Joint Committee on Cancer (AJCC) have collaborated on periodic revisions of this staging system, leading to the 7th edition in 2010.^4^

For gastric cancer, several changes to the 6th edition were made.^5^ In the 7th edition, all gastroesophageal junction (GEJ) tumors are staged as esophageal cancers, except tumors arising in the stomach >5 cm from the GEJ. The T classification categories have been redefined (Table 1), and the T classification of stomach cancer and esophageal cancer have been harmonized. N categories have been modified to better represent the distribution of the number of positive lymph nodes. The M1 category has been amended to include positive peritoneal cytology. Stage IV now includes only patients with M1 disease. Finally, new stage groups have been added to the staging system (IIB and IIIC). The 7th edition staging system is more complex, with an increase in the number of permutations of TNM groupings from 56 to 80. There are now nine stage groups, compared to seven in the AJCC 6th edition (Table 2).

**Table 1:** Changes in the AJCC staging system for gastric cancer

Tumor, node, metastases	AJCC 6th ed.	AJCC 7th ed.
T: Primary tumor		
Depth of invasion		
No evidence of primary tumor	T0	T0
Carcinoma-in-situ	Tis	Tis
Mucosa^a^	T1	T1a
Submucosa		T1b
Muscularis propria	T2a	T2
Subserosa	T2b	T3
Serosa	T3	T4a
Adjacent structures	T4	T4b
N: Regional lymph nodes		
No. of regional lymph node metastases		
0	N0	N0
1–2	N1	N1
3–6		N2
7–15	N2	N3a
>15	N3	N3b
M: Distant metastases		
No distant metastases	M0	M0
Distant metastases	M1	M1

^a^At least invasion of lamina propria

**Table 2:** Stage grouping according to the 6th and 7th edition AJCC staging system

6th edition AJCC staging system				7th edition AJCC staging system			
Stage	T	N	M	Stage	T	N	M
*0*	*Tis*	*N0*	*M0*	*0*	*Tis*	*N0*	*M0*
*IA*	*T1*	*N0*	*M0*	*IA*	*T1*	*N0*	*M0*
IB	T1	N1	M0	IB	T1	N1	M0
	*T2*	*N0*	*M0*		*T2*	*N0*	*M0*
II	T1	N2	M0	IIA	T1	N2	M0
	T2	N1	M0		T2	N1	M0
	T3	N0	M0		T3	N0	M0
				IIB	T1	N3	M0
					T2	N2	M0
					T3	N1	M0
					T4a	N0	M0
IIIA	T2	N2	M0	IIIA	T2	N3	M0
	T3	N1	M0		T3	N2	M0
	T4	N0	M0		T4a	N1	M0
IIIB	T3	N2	M0	IIIB	T3	N3	M0
					T4a	N2	M0
					T4b	N1	M0
					T4b	N0	M0
				IIIC	T4a	N3	M0
					T4b	N3	M0
					T4b	N2	M0
IV	T4	N1–3	M0	IV	*Any T*	*Any N*	*M1*
	T1–3	N3	M0				
	*Any T*	*Any N*	*M1*				

*T* tumor classification, *N* nodal status, *M* metastases status

*Italics* No changes in TNM and stage groups

With each staging system revision, there is a tension between improving prognostic value of the staging system by adding subdivisions of existing stage groupings and introducing new predictive parameters, and the desire to keep the staging system intuitive simple. The purpose of this study was to compare the 6th to the 7th edition of the AJCC staging system for gastric cancer, first by describing the differences in stage-specific survival, and second by examining whether the increased complexity of the 7th edition resulted in improved prognostic accuracy as compared to the 6th edition.

## Patients and Methods

The data set used for this study is a combination of two large prospectively collected databases.

### Memorial Sloan-Kettering Cancer Center

Between July 1985 and December 2009, a total of 2,589 patients with an adenocarcinoma of the stomach or GEJ underwent a gastrectomy at the Memorial Sloan-Kettering Cancer Center (MSKCC) and were entered in a prospectively maintained database. Patients with tumors of the GEJ (Siewert I–III, *n* = 669), and patients with a noncurative (R1 or R2) resection or with M1 disease (*n* = 358) were excluded. Because the data set focused on curative resections, all patients with M1 disease were excluded; all would have been staged as having stage IV disease in the 6th and 7th edition staging systems. Three patients with T0 N+ disease in their final pathology could not have a stage group assigned and were excluded, leaving 1,559 patients for analysis.

Most patients underwent a D2 lymph node dissection. Preoperative and postoperative therapy were administered according to the ongoing clinical trials and the standard of care at MSKCC during the study period. Adjuvant chemotherapy was provided infrequently from 1985 to 1999. From 2000 to 2009, perioperative chemotherapy became more common for advanced-stage tumors; postoperative chemoradiation was also administered between 2000 and 2007. Follow-up was generally conducted according to published National Comprehensive Cancer Network guidelines.^6^

Survival data were updated when available until March 2010. This study was approved by the institutional review board of MSKCC. This data set was used in part to help guide changes to the AJCC 7th edition.

### Dutch Gastric Cancer Trial

In the Dutch Gastric Cancer Trial (DGCT, 1989–1993), 1,078 patients with adenocarcinoma of the stomach were randomized for D1 or D2 lymphadenectomy.^7^–^9^ None of the patients had a tumor of the GEJ, while patients with metastatic disease (*n* = 367) and patients who underwent a noncurative resection (*n* = 74) were excluded, leaving 637 patients who underwent an R0 resection for this study. No adjuvant therapy was provided to these patients in the curative setting. Follow-up was conducted every 6 months. Recurrent disease was generally confirmed with radiology, endoscopy, and/or histology. Survival data were updated when available until November 2007.

### Staging

Because the Union for International Cancer Control (UICC) and AJCC use the same staging definitions, for purposes of clarity, the UICC/AJCC staging system is referred to here as the AJCC staging system. Tumor, nodal, and metastasis stage and stage grouping are all based on final postoperative pathology. All staging parameters (T, N, M) and stage groupings of the 6th and 7th edition staging system were calculated on the basis of depth of invasion through the gastric wall, the number of positive lymph nodes, and the presence or absence of distant metastases. No patients were excluded as a result of incomplete staging data.

### Statistical Analysis

Survival probabilities were estimated with the Kaplan-Meier method, and differences in survival curves were assessed by the log-rank test. The end point in this study was disease-specific survival, which was recorded from the date of surgery until the date of death of disease; dead from other causes and alive at last date of follow-up were recorded as censored events.

The concordance index between survival and stage for the two staging systems was calculated by a previously described methodology.^10^ Concordance for a staging system can range from 0 to 100 %, with 100 % representing absolute concordance, 50 % indicating no association (no better than flipping a coin), and 0 % perfect discordance. The concordance index for a staging system was calculated by analyzing all possible pairs of two patients in the data set. A pair of two patients is concordant if the patient with the higher stage has the shorter survival. Concordant pairs are assigned a value of 1, and discordant pairs are assigned a value of 0. The concordance of the staging system is the sum of the values of all the individual pairs divided by the total number of pairs in the data set. For pairs where the shorter survival time was censored, the stage-specific Kaplan-Meier estimate of survival was used. Pairs in which both patients were in the same stage group were assigned a value of 0.5. Therefore, the maximum concordance of the staging system could never be 100 %. The maximum potential concordance in our data set for the 6th edition was 0.818 and 0.853 for the 7th edition. Confidence intervals and *P*-values for the difference in concordance indices of the two staging systems were calculated by bootstrap resampling.

To validate the results provided by concordance analysis, the Brier score was used to evaluate the expected error of the predictions in both staging systems. For every patient, the Brier score measures the difference between the survival probability predicted by the staging system and the observed survival. Kaplan–Meier estimates were used for censored observations. The average squared deviation for all patients gives the Brier score, in which a lower score represents a better predictive accuracy.

## Results

### Patients

All 2,196 patients in this analysis underwent a radical (R0) resection for an adenocarcinoma of the stomach between July 1985 and December 2009, either at MSKCC (*n* = 1,559) or at one of the hospitals participating in the DGCT (*n* = 637). Patient characteristics are summarized in Table 3. Median follow-up was 98 months.

**Table 3:** Patient characteristics

Characteristic	Value	MSKCC (*n* = 1,559)	DGCT (*n* = 637)
	Total (*n* = 2,196)		
Gender			
Male	1,307 (60)	943 (61)	364 (57)
Female	889 (40)	616 (39)	273 (43)
Age	67 (22–96)	67 (22–96)	66 (31–84)
Location			
Proximal	630 (29)	525 (34)	105 (17)
Middle	630 (29)	430 (28)	200 (31)
Distal	899 (41)	572 (37)	327 (51)
Diffuse	37 (2)	32 (2)	5 (1)
Depth of invasion			
No tumor	35 (2)	31 (2)	4 (13)
Mucosa	231 (11)	150 (10)	81 (16)
Submucosa	355 (16)	255 (16)	100 (15)
Muscularis propria	282 (13)	189 (12)	93 (22)
Subserosa	464 (21)	322 (21)	142 (20)
Serosa	706 (32)	576 (37)	130 (14)
Adjacent organs	123 (6)	36 (2)	87 (1)
No. of evaluated nodes	21 (0–106)	21 (0–84)	22 (1–106)
Patients with at least 15 nodes evaluated	1,671 (76)	1,213 (78)	458 (72)
No. of positive nodes	1 (0–63)	1 (0–63)	1 (0–28)
Type of surgery			
Total gastrectomy	562 (26)	359 (23)	203 (32)
Proximal gastrectomy	106 (5)	106 (7)	0
Distal gastrectomy	1,222 (56)	788 (51)	434 (68)
Esophagogastrectomy	291 (13)	291 (19)	0
Wedge/sleeve resection	14 (1)	14 (1)	0
Unknown	1 (0.1)	1 (0.1)	0
Adjuvant therapy			
Preoperative chemotherapy	245 (16)	0	0
Postoperative chemotherapy	251 (16)	0	0
Postoperative radiotherapy	80 (5)	0	0

Because of rounding, the sum of the percentages is not always 100 %. Data are presented as *n* (%) or median (range)

### TNM Staging

**Fig. 1:**
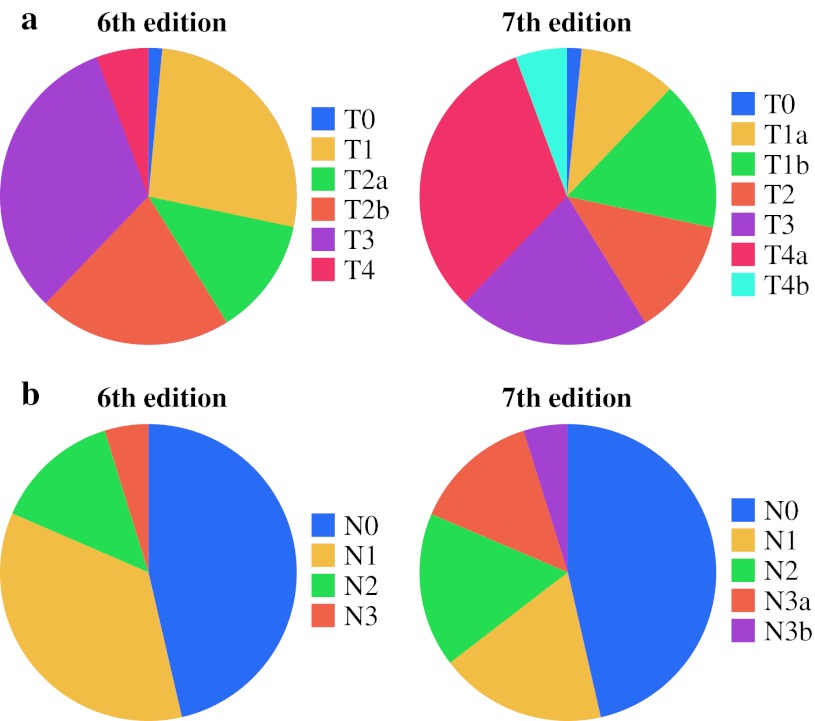
**a** Distribution of T classification in the 6th and 7th edition AJCC staging system (*n* = 2,196). In the 7th edition staging system, former category T1 is divided into new categories T1a and T1b, whereas other T categories are redefined. **b** Distribution of N classification in the 6th and 7th edition AJCC staging systems (*n* = 2,196). In the 7th edition staging system, former category N1 is divided into new categories N1 and N2, and category N3 is redefined

Figure 1 depicts the distribution of T and N classification of the 6th edition and 7th edition staging systems for all 2,196 patients. The redefined N1, N2, and N3 classifications were more evenly distributed. Among 2,196 patients, 674 (31 %) were assigned a higher N classification in the AJCC 7th edition. In the 7th edition staging system, the N3 category is divided into N3a (7–15 positive nodes) and N3b (16 or more positive nodes). This recognized the unique independent prognostic significance of an increasingly higher number of positive nodes, even at the high end.

### Stage Grouping

Differences in stage distribution between the two systems are listed in Table 4. For stage IB to IV, T, N, and M as well as stage group definitions were altered, leading to a change in stage group for 1,302 (59 %) of 2,196 patients. In total, 748 patients (34 %) moved to a higher-stage group, and 186 patients (9 %) moved to a lower-stage group. A total of 368 patients (17 %) with a stage II tumor in the 6th edition staging system were distributed between stage IIA and IIB in the 7th edition staging system. Of note, stage grouping did not make use of the N3a/N3b classification.

**Table 4:** Distribution of patients according to the 6th and 7th edition AJCC staging system

	7th ed.								Total
	0	IA	IB	IIA	IIB	IIIA	IIIB	IIIC	
6th ed.									
0	*35*								35
IA		*476*							476
IB			*220*	210					430
II				61	307	99			467
IIIA						*163*	258		421
IIIB								181	181
IV					1	1	44	140	186
Total	35	476	220	271	308	263	302	321	2,196

*Italics* patients who stay in the same stage group

### Discrimination Between Stage Groups

**Fig. 2:**
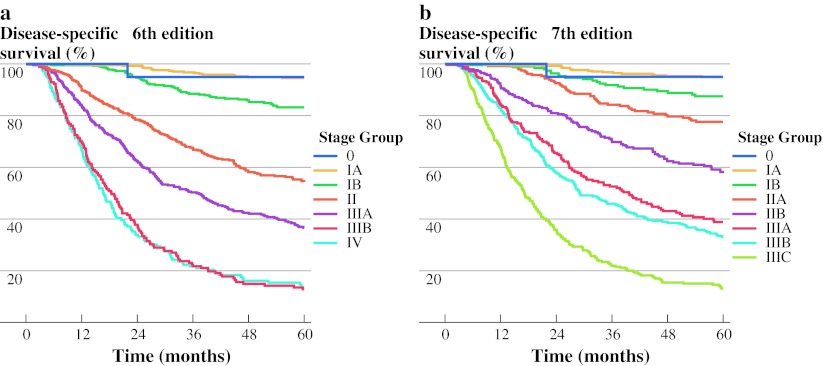
**a** Disease-specific survival according to the 6th edition AJCC staging system (*n* = 2,196). **b** Disease-specific survival according to the 7th edition AJCC staging system (*n* = 2,196)

Five-year survival estimates for both staging systems are shown in Table 5 and Fig. 2. In the 6th edition staging system (Fig. 2a), Kaplan–Meier survival estimates significantly differed for stage IA–IB, IB–II, II–IIIA, and IIIA–IIIB, but not for stage 0–IA (*P* = 0.64) and IIIB–IV (*P* = 0.60).

**Table 5:** Five-year and median disease-specific survival (DSS) estimates for stage groupings of the 6th and 7th edition staging system (*n* = 2,196)

Stage group	AJCC 6th edition		AJCC 7th edition	
	5-Year DSS (%)	Median DSS (mo)	5-Year DSS (%)	Median DSS (mo)
0	95.0	Not reached	95.0	Not reached
IA	94.6	Not reached	94.9	Not reached
IB	83.4	Not reached	87.5	Not reached
II	55.3	85		
IIA			77.5	278
IIB			57.6	119
IIIA	37.5	38	38.8	40
IIIB	14.0	19	32.9	29
IIIC			13.0	17
IV	14.4	17

In the new staging system, stage group 0 and IA remain unchanged. Differences between the 7th edition stage groups were significant for stage IA–IB, IIA–IIB, IIB–IIIA, and IIIB–IIIC but not for stage 0–IA (*P* = 0.64), IB–IIA (*P* = 0.09), and IIIA–IIIB (*P* = 0.15, Fig. 2b).

**Fig. 3:**
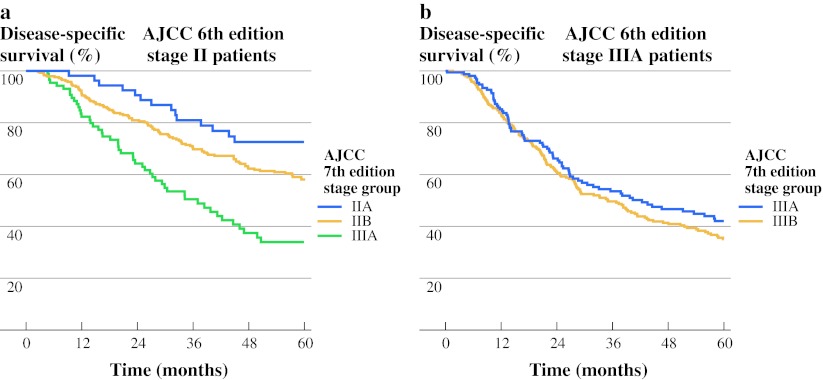
**a** AJCC 6th edition stage II patients (*n* = 467) are distributed between stages IIA, IIB, and IIIA in the 7th edition staging system. **b** AJCC 6th edition stage IIIA patients (*n* = 421) are distributed between stages IIIA and IIIB in the 7th edition staging system

Figure 3a shows patients from the 6th edition stage II, which is subdivided into stage IIA, IIB and IIIA in the 7th edition staging system. Differences between the curves were all significant. In Fig. 3b, the subdivision of 6th edition stage IIIA into 7th edition stage IIIA and IIIB is shown; no significant differences between the two new stage groups were detected (*P* = 0.26).

Overall, in the AJCC 6th edition, two out of six consecutive steps between stage groups were not significantly discriminant, while in the AJCC 7th edition, three out of seven consecutive steps were not significantly discriminant, indicating that the discriminant value between stage groups has decreased between the 6th and 7th edition staging system.

### Predictive Accuracy

The concordance index of T staging did not change significantly from the 6th to the 7th edition (*P* = 0.36) (Table 6). The concordance index of N staging showed an increase from 0.659 to 0.665 (*P* = 0.03). Despite the change in definition for almost every stage group and the increased number of stage groups, the concordance estimate for the 7th edition was 0.697, which was significantly inferior to that of the 6th edition staging system (0.711, *P* < 0.01). Brier score for T, N, and overall stage groupings showed no significant improvement from the 6th to the 7th edition.

**Table 6:** Predictive accuracy of the 6th and 7th edition AJCC staging system

Accuracy	AJCC edition	T classification	N classification	Stage group
Concordance	6th ed.	0.666 (*P* = 0.36)	0.659 (*P* = 0.03)	0.711 (*P* < 0.01)
	7th ed.	0.667	0.665	0.697
Brier score	6th ed.	0.165	0.165	0.158
	7th ed.	0.163	0.164	0.156

For concordance, higher is better. For Brier score, lower is better

## Discussion

The current study describes the impact of the changes made in the 7th edition of the TNM classification for stomach cancer by comparing stage-specific survival and predictive accuracy of the 6th and 7th edition staging system in a combined data set with over 2000 patients who underwent an R0 resection for gastric cancer.

Three earlier single-institution Asian studies compared the 6th and 7th TNM classifications for gastric cancer.^11^–^14^ The first study analyzed 9998 patients treated at a Korean university hospital and found a more detailed classification of prognosis in the 7th edition staging system, accompanied with increased homogeneity within stage groups.^11^ A Chinese study found better prognostic stratification in the 7th edition staging system.^12^ Another Korean study evaluated nodal classification in 295 patients and found that in multivariable analysis, N classification was an independent prognostic factor for survival in the 7th edition, but not in the 6th edition, staging system.^14^

One strength of the current study is the use of data from multiple institutions, thereby reducing the risk of unique outcome due to single-institution bias. However, both series are Western, and no Asian data set was used. Another advantage of the current study is the high quality of the data: all patients underwent an R0 resection, and disease-specific survival was used as the outcome measure. In the three previously published studies, overall survival instead of disease-specific survival was used, and in one study, 14.5 % of the patients underwent an R1 resection.^12^

With the redefinition of nodal classification, the distribution of patients among the N1, N2, and N3 categories is more equal (Fig. 1b), while the disease of many patients is upstaged under the new staging system. A point of discussion on nodal staging in gastric cancer is that in the Western world, lymph node yield is generally low, certainly in comparison with Asian centers.^15^,^16^ This leads to the potential shifting of patients into a more advanced nodal classification simply by investigating more lymph nodes.^17^ Several groups have suggested the use of lymph node ratio (metastatic/total lymph nodes) instead of nodal status because of its higher prognostic accuracy and the elimination of the effect of this shift.^18^–^20^ In these studies, however, cutoff values for lymph node ratio intervals are often based on the data set they used. This introduces an advantage for lymph node ratio, which will perfectly fit the data set the study uses, whereas TNM nodal classification is part of an established system. However, decreasing the threshold for N2 and N3 categories in the 7th edition staging system considerably reduces the shifting effect. A minimum number of 15 nodes, however, remains the recommended threshold for adequate nodal staging.

A limitation of the stage groupings of the 7th edition staging system is that N3a and N3b categories were combined as N3, thereby not recognizing the prognostic significance of having 7–15 positive nodes, versus more than 16 positive nodes in overall stage grouping. The introduction of N3a and N3b as separate categories in overall stage grouping will increase complexity of the staging system, but it is unknown whether it will improve overall predictive accuracy. This issue needs to be further addressed in future staging systems.

There are several benchmarks for comparing the performance of two staging systems. First, there should be homogeneity within stage groups; patients within the same stage group should have only small differences in survival. Second, there should be discrimination between stage groups; patients in different stage groups should have larger differences in survival. Third, a staging system should have good predictive accuracy; patients with a higher stage should have a worse survival. And fourth, a staging system should be as simple and intuitive as possible in clinical practice, because increased complexity impedes clinical utility.

### Homogeneity Within Stage Groups

Establishing homogeneity within stage groups requires grouping of TNM combinations that have similar survival estimates (Table 2). For homogeneity testing, results are highly dependent on the size of the data set. Ahn et al.^11^ showed improved homogeneity of two homogeneous stage groups in the 7th edition compared to one homogeneous stage group in the 6th edition, using a data set of nearly 10,000 patients. In the current study, numbers are smaller, and therefore significant homogeneity within stage groups is hard to detect (results not shown).

### Discrimination Between Stage Groups

Heterogeneity between stage groups can be assessed by comparing stage-specific survival estimates for significant differences. Whether differences between stage groups are significant is highly dependent on the size of the data set. Small differences in survival estimates between stage groups are more likely to be statistically significant in a large data set. In the current study, stage-specific heterogeneity has decreased in the 7th edition when compared to the 6th edition. Although AJCC 6th edition stage II contained a highly heterogeneous population (Fig. 3a), and distributing these patients between stages IIA, IIB, and IIIA in the 7th edition has created three groups with a significantly different prognosis, the distribution of 6th edition stage IIIA patients into AJCC 7th edition stages IIIA and IIIB has created two stage groups with almost identical stage-specific survival (Fig. 3b). Wang et al. showed decreased heterogeneity between stage groups in the 7th edition as well.^12^

### Prognostic Accuracy for Individual Patients

Performance of a staging system can also be assessed on the individual patient level by comparing survival of patients with different stages. Several ways of comparing staging systems on an individual-patient level have been proposed, but there is no standard method.^21^ Commonly used methods include explained variation (or Brier score), the area under the receiver–operator characteristic curve, the concordance index, and a summary measure of separation. We decided to use the concordance index and Brier score to measure the prognostic accuracy of the staging systems because they analyze different, complementary measures. Concordance index is a measure of whether ranking of patients by staging is consistent with the ranking of their outcome. Its advantages include interpretation (because it is a probability), robustness (because it is based on ranks, it is not sensitive to small changes in the data), and availability of appropriate statistical methods for estimation. It also incorporates a built-in penalty for staging systems with a higher number of categories, so that with equally performing staging systems, the system with more categories will have a lower concordance probability. It does not penalize possible shifts (miscalibrations) between predicted and observed survival. Therefore, we also used the Brier score because it looks at the actual difference (in months) between predicted and observed survival, taking possible shifts into account.

In the current data set, concordance analysis showed no difference for T category, an improvement for N category, and a decline for stage grouping. Brier scores consistently showed no significant improvement from the 6th to the 7th edition. Therefore, it can be concluded that for individual patient outcome, no improvements were detected from the 6th to the 7th edition staging systems.

Only one of the previously published studies compared the two staging systems on an individual-patient level. It found increased predictive accuracy for the 7th edition staging system.^12^ A disadvantage of the method employed in that study is that the metric used for comparison, the Akaike information criterion, measures how well the staging system fits to the used data set without assessing the actual prognostic accuracy.

### Complexity of the Staging System

The larger number of stage group categories for the 7th edition of the staging system means that the system has become more complex. Increasing the number of categories of the staging system is not unique to gastric cancer.^4^ With the increasing availability of pathologic and molecular data, there is a trend toward incorporating more and more information into newer staging systems. Although these new categories might better reflect the natural history and prognosis of these diseases, there is a limit to the improvement of prognostic accuracy achievable with a categorical anatomic-based staging system like the TNM classification.^22^,^23^ At the same time, the goal of creating an intuitive, easy-to-use staging system disappears, and in daily clinical practice, cancer staging consists of using complex tables, if it is used at all.

Meanwhile, tools for individual patient prognostication have been developed that significantly outperform the TNM classification in prognostic accuracy. For gastric cancer, a nomogram has been developed based on a single US institution’s database.^24^,^25^ This nomogram has been validated in several international patient cohorts.^26^–^28^ The question is whether the TNM classification should aspire to the same goal of highly accurate individual patient prognostication as these nomograms. Prognostication is only one of the five goals of the TNM classification; all the other goals are directed toward a simple, intuitive international language: to aid the clinician in planning and evaluating treatment, to facilitate the exchange of information, and to contribute to research.^1^

In summary, the 7th edition of the AJCC staging system for gastric cancer has resulted in improved predictive accuracy for the N classification but decreased heterogeneity among stage groups. The increased complexity of the 7th edition staging system is not accompanied by an improvement in prognostic accuracy of stage grouping. Staging represents a compromise in accounting for the most reproducible and prognostically relevant factors to aim at a simple, intuitive, useful, common language to describe the natural history of a tumor. It should not be confused with more complex multivariable prognostication models, which may be useful in defining groups of patients at homogenous risk of recurrence, regardless of anatomic TNM characteristics.
